# The Rare Condition of Left Ventricular Non-Compaction and Reverse Remodeling

**DOI:** 10.3390/life13061318

**Published:** 2023-06-03

**Authors:** Cristiana Bustea, Alexa Florina Bungau, Delia Mirela Tit, Diana Carina Iovanovici, Mirela Marioara Toma, Simona Gabriela Bungau, Andrei-Flavius Radu, Tapan Behl, Adrian Cote, Elena Emilia Babes

**Affiliations:** 1 Department of Preclinical Disciplines, Faculty of Medicine and Pharmacy, University of Oradea, 410073 Oradea, Romania; cristianabustea@yahoo.com; 2 Doctoral School of Biomedical Sciences, University of Oradea, 410087 Oradea, Romania; diana_iovanovici@yahoo.com (D.C.I.); mire.toma@yahoo.com (M.M.T.); sbungau@uoradea.ro (S.G.B.); andreiflavius.radu@gmail.com (A.-F.R.); 3 Department of Pharmacy, Faculty of Medicine and Pharmacy, University of Oradea, 410028 Oradea, Romania; 4 School of Health Sciences & Technology, University of Petroleum and Energy Studies, Bidholi, Dehradun 248007, India; tapanbehl31@gmail.com; 5 Department of Surgical Disciplines, Faculty of Medicine and Pharmacy, University of Oradea, 410073 Oradea, Romania; adrian.cote@didactic.uoradea.ro; 6 Department of Medical Disciplines, Faculty of Medicine and Pharmacy, University of Oradea, 410073 Oradea, Romania; babes.emilia@gmail.com

**Keywords:** left ventricular non-compaction, heart failure, reverse remodeling, cardiomyopathy, cardiac magnetic resonance imaging, angiotensin receptor neprilysin inhibitor, ARNI therapy

## Abstract

Left ventricular non-compaction (LVNC) is a rare disease defined by morphological criteria, consisting of a two-layered ventricular wall, a thin compacted epicardial layer, and a thick hyper-trabeculated myocardium layer with deep recesses. Controversies still exist regarding whether it is a distinct cardiomyopathy (CM) or a morphological trait of different conditions. This review analyzes data from the literature regarding diagnosis, treatment, and prognosis in LVNC and the current knowledge regarding reverse remodeling in this form of CM. Furthermore, for clear exemplification, we report a case of a 41-year-old male who presented symptoms of heart failure (HF). LVNC CM was suspected at the time of transthoracic echocardiography and was subsequently confirmed upon cardiac magnetic resonance imaging. A favorable remodeling and clinical outcome were registered after including an angiotensin receptor neprilysin inhibitor in the HF treatment. LVNC remains a heterogenous CM, and although a favorable outcome is not commonly encountered, some patients respond well to therapy.

## 1. Introduction

Left ventricular non-compaction (LVNC), also known as spongiform cardiomyopathy (CM), is a rare heart muscle disease that is due to the failure of myocardial compaction in the first trimester of fetal development. The ventricular wall in LVNC has two layers: a thick, hyper-trabeculated myocardium layer and a thin, compacted epicardial layer. After conducting autopsy research on a newborn with the congenital abnormalities of a coronary ventricular fistula and aortic atresia, Bellet and Gouley published the first description of LVNC in 1932. Isolated non-compaction CM was first described in an echocardiographic study performed by Engberding and Bender in 1984 [1].

There are controversies regarding whether LVNC is a distinct CM or a phenotypic manifestation of various cardiomyopathies [2]. The criteria for diagnosis are mainly based on the morphological changes detected in non-invasive imaging. The most commonly used diagnostic methods are echocardiography, cardiac magnetic resonance (CMR), and computed tomography [3]. Although the American Heart Association classifies LVNC as a main genetic CM [4], the pattern of non-compaction of the LV can be observed in several clinical situations. A persistent area of debate is the differentiation from normal LV trabeculation or the relationship with cardiomyopathies such as dilated or hypertrophic cardiomyopathy, which may share the same genetic basis.

The genetic pattern and clinical presentation are variable. The clinical picture is heterogeneous, but the main manifestations consist of heart failure (HF), thromboembolic events, and ventricular arrhythmias. Regarding genetic transmission, LVNC is usually autosomal dominant, but an autosomal recessive X-linked or mitochondrial inheritance type may be present [5]. LVNC may appear isolated or in association with other cardiac pathologies, and some cases appear in patients with neuromuscular diseases [3] or in patients with metabolic abnormalities [5,6,7]. Because it is currently uncertain whether LVNC is a distinct condition or a morphological characteristic that is influenced by other cardiomyopathies, the European Society of Cardiology’s group of specialists on pericardial and myocardial illness includes it in the undefined cardiomyopathies category [8].

The therapeutic approach in LVNC is not well studied. For LVNC with reduced ejection fraction, therapeutic strategies were extrapolated from patients with dilated CM. The current guideline recommendation should be followed for HF treatment in LVNC. Previous reports have shown that some cases of LVNC and LV systolic dysfunction can develop reverse remodeling after optimal therapy [9,10,11,12]. Reverse remodeling in LVNC is associated with improved outcomes.

The purpose of the present study is to expose the therapeutic and diagnostic challenges of this rare disease. HF represents the most common presentation in LVNC, and there are no specific studies or recommendations for HF treatment in this type of patient. The novelty presented by the case that is provided as an example is the important improvement achieved after the optimization of HF treatment by adding angiotensin receptor neprilysin inhibitor (ARNI) to the therapy; this phenomenon has only been described in a few cases in the literature. For this study, published data from 1984 to 2023 on the topic were searched in the most known databases using the keywords mentioned at the beginning of the present paper.

## 2. Epidemiology and Pathogenesis

LVNC is a rare disease with a prevalence that is difficult to estimate, as the criteria for diagnosis are not uniformly defined. The current data report a prevalence of 0.01–0.3% in the adult population, with male predominance [13,14].

The myocardium presents intertrabecular recesses and a trabeculated structure during the formation of the heart. During the first trimester of pregnancy, the ventricular muscles undergo compaction to form a solid myocardial layer, and the intertrabecular recesses become the coronary arteries. Early impaired LV compaction during embryonal endomyocardial morphogenesis will result in the development of a compacted epicardial stratum and an endocardial stratum with pronounced trabeculae and profound intertrabecular recesses that connect to the LV cavity. Recently, some other opinions have emerged; Jensen et al. suggested that hyper-trabeculation in LVNC may result from the compacted myocardium growing into the ventricular lumen in a trabecular fashion [15]. Ventricular non-compaction is most commonly located at the apex of the heart due to the fact that the compaction process not only progresses from the epicardium to the endocardium, but also from the base toward the apex of the heart [16]. More than 80% of patients have the apical, mid-inferior, mid-lateral, and mid-anterior wall segments involved [3,17].

Right ventricle involvement may also be present during hyper-trabeculation, dilatation, and dysfunction [18]. A differential diagnosis between a pathologically non-compacted myocardium and increased trabeculations representing a normal variant depends on the existence of a thinner compacted myocardial wall in LVNC and a normal thickness of the compacted myocardial layer in the normal variant [13].

LVNC is usually associated with systolic dysfunction and a reduced ejection fraction. Hypokinesia of the compacted and non-compacted layers and the asynchronous contraction between the compacted and non-compacted myocardial layers will determine systolic dysfunction. Although epicardial coronary arteries are normal, subendocardial hypoperfusion may be present due to a discrepancy between the cardiac mass and the amount of capillaries and microcirculatory dysfunction. Progressive fibrosis determined by ischemia will depress LV systolic function and favor ventricular arrhythmias. Ventricular compliance is also affected by trabeculations, resulting in diastolic impairment. Hyper-trabeculations determine irregular relaxation, constrictive filling, and diastolic dysfunction [17,19].

## 3. Etiology

Regarding etiology, the most common form of LVNC is sporadic; however, it can also be familial, having autosomal dominant inheritance [18]. LVNC’s existence has been associated with several genes, including more frequent sarcomere genes (82%) [20]. Genes that encode cellular signaling networks, such as sarcomere proteins, and ion channels that have been associated with LVNC are implied as well in dilated and hypertrophic CM [18]. LVNC may appear in association with other cardiomyopathies, including dilated CM, restrictive CM, hypertrophic CM, congenital heart diseases (Ebstein disease), or arrhythmogenic right ventricular CM [21]. LVNC can also be found to be associated with Barth syndrome, neuromuscular diseases, or metabolic diseases [5].

LV dysfunction is more common in genetic cases rather than in sporadic cases, and this is usually an indicator of a worse outcome [20]. The genes *MYBPC3*, *MYH7*, and *TTN* are the ones that are most often involved [21]. A genetic variant of *MYH7* seems to be associated with biventricular disease and a restrictive filling pattern of diastolic dysfunction [22]; alternatively, it is associated with a significant systolic dysfunction that is associated with a dilated phenotype [23] needing urgent transplant. Genetic testing has a good genetic yield and is useful for prognostic estimation, as patients without underlying an genetic cause have a better outcome [24].

When LVNC is identified, family screening may be beneficial in the evaluation of familial cases. First-degree relatives should be assessed as they can be affected in 13–50% of cases [5]. For family members with LVNC or trabeculations, close surveillance is recommended [25].

## 4. Clinical Presentation

The clinical presentation and morphological expression of LVNC are highly variable. Due to challenges in the diagnosis, according to the French LVNC records, there is a mean diagnosis delay of 6.43 ± 3 years, and 32% of patients are diagnosed with this impairment after 5 years or later [26].

The average age of patients (at diagnosis) might range from 37 ± 17 years [24] to 45 ± 17 years [27]; the majority of patients are symptomatic at the moment of diagnosis. HF is the main presentation form in up to 62% [17,25] of instances, and 98% of black people are affected [28]. Nearly 50% of subjects are in New York Heart Association (NYHA) classes III–IV at presentation. Aside from HF, thromboembolic events and arrhythmias are common clinical manifestations. Embolic events are the results of thrombus formation in the non-compacted myocardium deep clefts between trabeculations and are described in 5–38% of patients [17].

Arrhythmias are frequent in LVNC. Ventricular tachycardia occurs in 38–47% of cases because of a substrate that typically involves the non-compacted mid-apical LV segments. Ventricular tachycardia and fibrillation are more common in patients with severely reduced systolic function. Over 25% of cases have been reported for atrial fibrillation. Complete heart block and paroxysmal supraventricular tachycardia may also appear [17]. WPW syndrome was observed in 1.5% of cases [3]. QTc prolongation is observed in over 50% of patients, and early repolarization abnormalities are common [17].

## 5. Diagnostic Approach

The diagnosis is based on imagistic methods, with echocardiography and CMR imaging being the most used. Although the first echocardiographic diagnosis was published more than 30 years ago, there are still no generally accepted diagnostic criteria [3].

### 5.1. Echocardiography

The echocardiographic criteria proposed by Jenni are the most widely accepted and were confirmed in our patient. The parasternal short-axis view supports LVNC when the non-compacted layer/compacted layer end systolic ratio is bigger than two. The absence of other abnormalities of the heart and the presence of a color flow Doppler in the deep intertrabecular recesses are additional criteria for diagnosis [29].

Other echocardiographic criteria are those used by Chin et al., who defined LVNC as the proportion of compacted/compacted + non-compacted myocardium < 0.5 at end-diastole in the parasternal short-axis perspective of the apex and in the apical views for the LV free wall [30]. Stollberg’s echocardiographic definition for LVNC is the presence of trabeculations (>3 trabeculae) seen in one imaging plane and apically extending from the LV wall towards the papillary muscle in end-diastole, synchronously moving with the compacted myocardium [31]. Only 30% of patients fulfill all three criteria. Other echocardiographic techniques are recommended for challenging cases. Speckle tracking echocardiography is useful in borderline cases because in LVNC, the LV twist is affected. Speckle tracking can identify the direction of the basal and apical rotations. Dalen et al. presented this abnormal rotation pattern in patients with LVNC, which was characterized by LV solid body rotation, with basal and apical rotation oriented in the same direction and almost no LV twist [32]. A reduced global longitudinal strain is a sensitive sign of systolic dysfunction [11]. Three-dimensional echocardiography analysis can evaluate the extent of the non-compacted layer and contribute to the diagnosis of patients with LVNC. Additional contrast in echocardiography is useful, according to the European Association of Cardiovascular Imaging’s recommendations [33], and can better delineate endocardial borders, trabeculations, and the perfused intertrabecular recesses [34].

LV systolic dysfunction is common but not mandatory in subjects suffering from LVNC. The gravity of the disease is associated with LV dysfunction severity. The degree of LV dysfunction was found to be associated with the extent of myocardial non-compaction by some authors [35]. The amount of damaged segments and the ratio of non-compacted/compacted myocardium appear to be the main predictors of LV systolic malfunction, while left ventricular ejection fraction (LVEF) remains an important predictor of mortality [36]. In contradiction with these results, an Italian study on 238 consecutive patients with LVNC revealed that the amount of non-compacted segments does not appear to have a connection to ventricular dysfunction [37].

Another study that compared patients with isolated forms of LVNC to patients with dilated CM and prominent trabeculations revealed that the end-diastolic volume index and LV sphericity index were considerably lower in individuals with isolated LVNC; however, the patients had more trabeculated segments, a higher non-compacted/compacted myocardium ratio, and a significantly higher LVEF. The stroke volume index, cardiac output, and cardiac index were similar in patients with isolated forms of LVNC and those with dilated CM. The ratio of non-compacted to compacted myocardium and the amount of trabeculated segments were directly correlated with LV end-diastolic volume index and inversely correlated with LVEF in subjects with isolated forms of LVNC. The amount of non-compacted segments and the non-compacted/compacted myocardium ratio were not significantly correlated with the LV end-diastolic volume index or with LVEF in patients with dilated CM [38].

It seems that the systolic dysfunction of the LV is closely correlated with the location and severity of the abnormal myocardial segments and the electro-mechanical activation of these areas; it is less correlated with the number or ratio of non-compacted/compacted myocardium [39,40]. Mitral annulus enlargement and mitral regurgitation were similar in the LVNC and dilated CM patients, and there was no correlation between the number of non-compacted segments and mitral annulus diameter or area [41].

### 5.2. Cardiac Magnetic Resonance

Although currently, the diagnosis gold standard is echocardiography, in many cases, it is necessary to perform a multimodal imagistic evaluation (echocardiography and CMR). CMR imaging is superior for identifying non-compacted myocardium and trabeculations, particularly at end-diastole [42], and it allows for a non-invasive tissue evaluation, quantification of the extent of non-compacted myocardium, and detection of segmental non-compaction in any area of the LV wall. Coexistent right ventricular non-compaction can be better identified using CMR, and delayed enhancement imaging can visualize myocardial fibrotic areas that correlate with the severity of LV dysfunction [43] and may represent the substrate for potentially lethal arrhythmias, modifying the treatment of these patients by the preventive insertion of an internal cardioverter defibrillator [44].

Jacquier et al.’s criteria define LVNC based on the magnitude of the trabeculated mass. A specific and sensitive sign for the CMR identification of LVNC is a non-compacted mass that represents more than 20% of the global LV mass at end-diastole [45]. The CMR criteria of Peterson et al. for diagnosis are a non-compacted/compacted myocardium ratio > 2.3 in a long-axis end-diastolic image in at least two consecutive segments [42]. Nucifora et al. observed that in patients with LVNC, trabeculations are predominantly located on the apex, anterolateral, and inferolateral walls; systolic dysfunction of the LV was encountered in half of the cases, and more than half of the patients had mid-wall late gadolinium enhancement (LGE) [18].

In a multicentric prospective study, Andreini et al. [46] found that LV dilatation, systolic dysfunction, and late gadolinium enhancement are independent predictors of poor outcome. Although the degree of trabeculation did not have a significant prognostic impact, it seems that LV trabeculation is correlated with reduced myocardial deformation indexes [13]. Native T1 mapping can detect diffuse myocardial fibrosis earlier than LGE [47].

Dodd et al. [48] found a higher grade of delayed myocardial enhancement during CMR processing in patients with more advanced disease progression. In a retrospective study on 75 patients with LVNC that were evaluated with a CMR examination, echocardiography, and subsequent clinical follow-up, it was observed that mitral regurgitation was frequent in LVNC with LV dysfunction. In patients with severe MR, the LV remodeling was worse, and the coexistence of LGE was associated with a poorer outcome. Both fibrosis and moderate–severe mitral regurgitation are related to the occurrence and development of myocardial maladaptive remodeling, and they have a combined effect in worsening the outcome [49]. Recent studies have revealed the role of LGE findings in risk stratification. Although LGE seems to be less frequent in LVNC compared with other cardiomyopathies [50], it was reported in many studies to be an important predictor of major cardiac events, with added prognostic implications over LVEF [43,46,51,52]. Myocardial fibrosis detected by LGE may be a consequence of coronary microvascular dysfunction and decreased coronary flow reserve; it can be followed using adverse remodeling and severe HF, but it is also associated with severe arrhythmias and adverse outcomes [53,54]. A systematic review conducted by Grigoratos et al. [55], which included four studies, further confirmed that the presence of LGE in LVNC was associated with multiple adverse outcomes, including cardiac death. On the other hand, a negative LGE and preserved LVEF were the factors that were associated with a good prognosis.

These findings sustain the use of CMR in the routine assessment of patients with LVNC. LGE for the detection of fibrosis should be part of the evaluation and may influence treatment decision guidance with respect to the risk of sudden cardiac death [56].

The pattern of LGE provides supplementary information. A multicenter study that evaluated the prognostic role of LGE in LVNC observed an increased risk of major cardiac events when the size of the LGE exceeded 7.5%, was ring-like, had many segments, and involved the free wall [57].

It is obvious that patients with LVNC and a positive LGE have more maladaptive LV remodeling and a higher incidence of adverse cardiovascular events; the absence of LGE is associated with LV reverse remodeling and a good prognosis, especially if LVEF is preserved.

### 5.3. Computed Tomography

Contrast-enhanced computed tomography can describe the abnormal architecture of the non-compacted LV wall and assess ventricular function. The spatial resolution of cardiac computed tomography for the identification of LVNC is good and permits visualization of the coronary arteries and great vessels; also, it is able to exclude coronary artery disease in subjects that present a low likelihood of ischemic heart impairment [58,59].

LVNC may manifest alone as a morphological phenotype, can be linked to LV systolic dysfunction and dilation, or can be associated with LV hypertrophy. In MOGE(S) nosology, pure LVNC (MLVNC) and LVNC with LV hypertrophy (MLVNC-H), and LV dilatation and dysfunction (MLVNC-D) are distinguished [60].

## 6. Therapeutic Approach

There is no specific therapy for LVNC, as treatment is targeted at clinical manifestations: HF, arrhythmias, and systemic embolism prevention.

### 6.1. Treatment of Heart Failure and Evidence for Reverse Remodeling Therapies

Patients with HF should be managed according to the current guidelines [61]. During the progression of HF, there is an increased stimulation of the cytokine and neurohormonal structures with the consequence of an increased alteration of myocytes, myocyte loss, alterations in the extracellular matrix, and changes in LV chamber geometry. Treatments that can reverse this remodeling will determine a functional improvement [62].

ARNI therapy in patients with LVNC is reported in a limited number of cases [63,64]. Sacubitril/Valsartan therapy in an individual with LVNC was linked to an amelioration of the clinical and echocardiographic markers, as reported by Bonatto et al. This patient with LVNC presented HF and underwent standard medical treatment in accordance with the guidelines for 18 months; however, there was not an enhancement in the patient’s clinical or echocardiographic markers. A considerable modification of the NYHA class (from III to I) along with considerable ventricular reverse remodeling followed the commencement of sacubitril/valsartan medication [63]. Another recent case study was reported concerning a patient with a dilated subtype of LVNC who had a spectacular improvement in LV systolic and diastolic function and important LV reverse remodeling with ARNI therapy [65]. PROVE HF (prospective investigation of ventricular remodeling, symptom relief, and biomarkers throughout Entresto therapy for HF) and EVALUATE HF (study comparing the effects of sacubitril/valsartan and enalapril on aortic stiffness in individuals with mild to moderate HF and a low ejection fraction) showed that ARNI-treated patients experienced positive LV remodeling, which was accompanied by a drop in NT-proBNP levels and clinical improvement; however, LVNC patients were not included in these investigations [66,67]. Furthermore, even in a short-term evaluation, ARNI appears to ameliorate LV size and hypertrophy more than angiotensin-converting enzyme inhibitors/angiotensin receptor inhibitors, as concluded in a recent meta-analysis on patients with HF and reduced ejection fraction [68].

The underlying mechanism of reverse remodeling remains partially unclear. The simultaneous inhibition of neprilysin and of the renin angiotensin aldosterone system using sacubitril/valsartan will result in a more effective neurohormonal modulation. By inhibiting neprilysin, an enzyme has a role in the degradation of natriuretic peptides in circulation, and all of the favorable effects of the circulating natriuretic peptides will be preserved. Vasoconstrictors angiotensin II and endothelin-1 are additionally divided by neprilysin, but the harmful effects of angiotensin II on the vascular system and heart are inhibited by valsartan [69]. Neprilysin inhibition may also influence the circulating levels of other peptides, which may additionally contribute to favorable effects of sacubitril/valsartan. Moreover, neprilysin cleaves apelin [70], and as a consequence of ARNI therapy, the level of apelin may increase, promoting angiotensin-converting enzyme 2 expression, stimulating the formation of vasodilating substrates, and antagonizing angiotensin II [71].

Beyond the impact on the renin–angiotensin–aldosterone system and the natriuretic peptide system, sacubitril/valsartan is reported to reverse cardiac remodeling. In the PROVE-HF trial, improved markers of cardiac function, volume decrease, and a reduction in the circulating levels of NT-proBNP were reported in subjects with HF with decreased ejection fraction that underwent treatment using sacubitril/valsartan [66]. Reverse LV remodeling with substantially enhanced ventricular volume overflow and dimension parameters, which subsequently determined the increase of LVEF, was reported in multiple other studies [72,73].

The cellular and molecular mechanisms of reverse remodeling in sacubitril/valsartan therapy are complex and still not completely understood. Sacubitril/Valsartan enhances myocardial calcium homeostasis, which helps promoting heart function [74] and may modulate proteins such as cysteine-rich protein 3 and titin, which participate in force transmission within the sarcomere [75].

Sacubitril/Valsartan affect cardiac structure and have an antihypertrophic effect that is not correlated with a blood pressure reduction. There are multiple mechanisms involved in the protective antihypertrophic effect. The two drugs act in synergy to prevent cardiomyocyte cell death and matrix remodeling. The combination of drugs blocks the activation of extracellular signal-regulated kinase that has an essential function in the pathogenesis of cardiac hypertrophy, and the combination also inhibits the angiotensin II receptor pathway. The molecular processes of the remodeling action of ARNI were described in a recent report. Valsartan inhibits proteins from the guanine nucleotide-binding complex, and sacubitril improves myocardial contractility and reduces myocardial cell death and hypertrophy [76]. In addition, sacubitril/valsartan suppresses several other signaling routes that are engaged in matrix remodeling, cardiac fibrosis, and apoptosis.

By blocking the TGF-1/Smad3 and Wnt/β-catenin signaling pathways, sacubitril/valsartan reduces cardiac fibrosis. Other signaling pathways such as the phosphatidylinositol 3-kinase/protein kinase B/glycogen synthase kinase-3β (PI3K/Akt/GSK-3β) and hypoxia-induced mitogenic actor (HIMF)-IL-6 may be influenced by sacubitril/valsartan, but further investigations are required to define them. These networks are involved in controlling cardiac fibrosis [77].

Mitochondrial energy production is increased by sacubitril/valsartan, leading to improved myocardial contractility via a SIRT3-dependent pathway. The effects of sacubitril/valsartan on nuclear respiratory factor-1 (NRF-1), nuclear respiratory factor-2, and mitochondrial transcription factor A needs further investigations [78].

Sacubitril/Valsartan antihypertrophic benefits are generally attributed to its potential to lessen extreme oxidative stress and inflammatory responses, which eventually slow down the remodeling process. Further investigations are required regarding the substance modulating actions on nuclear factor erythroid 2-related factor 2 (Nrf2)/antioxidant responsive element (Nrf2/ARE) signaling route, as well as how the substance acts on Kelch-like ECH-associated protein 1 (Keap1) [69]. In HF models, sacubitril/valsartan reduced the production of oxidative products, intracellular reactive oxygen species (ROS) such as inflammatory factors (IL-1β, interleukins IL-6, and TNF-α), and malondialdehyde [79]. In HF patients, a reduced oxidative stress and inflammation was revealed during therapy with sacubitril/valsartan [80,81].

Furthermore, there are some data suggesting that sacubitril/valsartan determines the better LV remodeling and outcome in subjects suffering from non-ischemic HF compared with those with ischemic HF [82,83].

Favorable remodeling, the improvement of systolic function, and the reduction of LV end-diastolic dimensions in LVNC after optimal therapy with medication or devices with known reverse remodeling potential were observed in a few studies and case reports (Table 1).

Therapy with at least one medication, such as beta-blockers, ACE inhibitors, or angiotensin receptor blockers, had a favorable effect in many young patients with the LVNC dilated phenotype, as evaluated in a retrospective study. There was a significant increase in the ejection fraction and shortening fraction (*p* < 0.0001) and a decrease in the LV end-diastolic dimensions (*p* < 0.05). Early diagnosis and medical treatment of LVNC can produce favorable LV remodeling [12]. Therapeutic approaches for patients with echocardiographic criteria for LVNC and reduced EF according to current HF guidelines resulted in several cases of the regression of LV hyper-trabeculations and an improvement in LV systolic function, which are associated with a better prognosis [87].

Bertini et al. concluded that the impact of cardiac resynchronization treatment on LV reverse remodeling in LVNC individuals and dilated CM are greater than in patients with dilated CM. By using standard and contrast echocardiography techniques, the authors observed a better response and greater LV reverse remodeling in those with a greater region of non-compaction. The amount of LVNC segments had a trend towards reduction compared with the baseline (*p* = 0.067), and patients with more trabeculated segments at baseline (>4) were more likely to be responders or super-responders (*p* = 0.003) [86].

Mechanical desynchrony is common in patients with LVNC, is independent from QRS width, and is correlated with the impaired electrical endocardial activation associated with the abnormal myocardium, which could justify an extended indication of biventricular pacing in this population. When analyzing the response to cardiac resynchronization therapy for individuals with LVNC as opposed to those with other cardiomyopathies by using gated-SPECT myocardial perfusion imaging, it was observed that cardiac resynchronization therapy contributes to an important improvement in patients with non-compaction myocardium. Desynchrony was assessed by determining the phase standard deviation, and patients with LVNC with more important desynchrony at baseline had the most significant improvement in intraventricular synchronism. The living standard improved in all patients, but non-ischemic subjects with and without LVNC had the most important improvements in LVEF and LV volume reduction [84].

Another study revealed that cardiac resynchronization therapy can improve the ejection fraction (*p* < 0.01), morphology, and mechanical desynchrony in LVNC patients. This study evaluated LV remodeling and mechanical synchronicity before/after 6 months of cardiac resynchronization therapy in LVNC patients. The LV reaction was established as a ≥15% reduction in the LV end-systolic volume. A percentage of 33.3% responded to cardiac resynchronization therapy, and they were super-responders (reduction in LVESV > 30%). All three desynchronies (inter-ventricular, radial intra-ventricular, and longitudinal) and both the non-compacted to compacted myocardium ratio and quantity of non-compacted segments decreased (for all *p* < 0.05) [85].

In a systematic review of the literature that included 14 studies, the authors concluded that cardiac resynchronization therapy can provide beneficial effects, improving clinical status and LVEF in LVNC patients with HF. A more important LV reverse remodeling was observed in cardiac resynchronization therapy responders, and this therapy is able to improve the performance of LVNC segments [86].

Indications for CRT in LVNC remain the same as in other cardiomyopathies: symptomatic HF patients, despite optimal medical treatment and in sinus rhythm, have an LVEF ≤ 35% and QRS duration ≥150 ms (class I level of evidence A) or between 130 and 149 ms (class IIa level of evidence B), mainly with a left bundle branch block morphology [88]. In addition, left bundle branch block is a frequent anomaly in LVNC that is reported in 41.7% of patients, as observed by Akhbour et al. [89]. A slightly higher incidence of left bundle branch block and a greater mean QRS width (although not statistically significant) are registered in this population compared with other cardiomyopathies [84].

Lin et al. reported a patient with a positive response to standard HF therapy, which is rarely encountered in patients with documented LVNC [90]. Stöllberger et al. reported the regression of LV hyper-trabeculation after improvement of LV systolic function with biventricular pacing. A compensatory mechanism of the failing heart was suggested as the etiology of LV hyper-trabeculation in this patient [10]. Cortez-Dias et al. described a case of a young black male with LVNC, severe LV systolic dysfunction, and severe aortic regurgitation who had a family history, possibly indicating a hereditary disorder; his case had an excellent evolution after aortic valve replacement [9]. Eurlings et al. presented a case of isolated LVNC treated with medical therapy and internal cardioverter defibrillator implantation that became less clear after 6 months of treatment [91], and another case was described by Luckie et al. using the resolution of echocardiographic features of LVNC in 18 months after standard medical therapy [92].

Vinardell et al. described the case of a woman with isolated LVNC who was managed by a guidelines-determined medical treatment, implantation of an internal cardioverter defibrillator, and resynchronization treatment; her complications resolved 2 years after the initial diagnosis with a normalization of LV volumes and ejection fraction and an almost complete resolution of the previously noted trabeculations. The authors concluded that non-compaction CM can either have a dynamic course or may be reversible, or that current morphologic criteria may occasionally misclassify a transient CM as non-compaction [93].

A non-compaction that is sometimes a partially reversible phenotype can be observed in athletes, pregnant women, and chronic HF patients, probably as an adaptative reaction to ventricular overload [94]. It is an area of debate whether a hyper-trabeculated LV without symptoms, LV dysfunction, or a family history of LVNC can be viewed as the initial stage of CM. Zemrak et al. followed up on 2742 asymptomatic subjects without known cardiovascular disease (CVD) that fulfilled the CMR criteria of LVNC at baseline for 10 years and observed that this morphological change appeared to be benign and was not linked with the deterioration of LV volumes or function over time [95]. LVNC includes a very large range, ranging from entirely morphological features with good a prognosis to a real muscular disorder with a possible adverse outcome [34].

### 6.2. Arrhythmias and Systemic Embolism Prevention

Anticoagulants are recommended in patients with LVEF ≤ 40%, atrial fibrillation, intracardiac thrombi, or previous embolic events [58]. Patients with malignant ventricular tachyarrhythmia necessitate the implantation an internal defibrillator as a supplementary measure to prevent sudden cardiac death. Radiofrequency ablation may be considered. Subjects with LVNC, CM, and an ejection fraction ≤ 35% have an indication for the insertion of an internal cardioverter defibrillator as an essential measure against unexpected cardiac death [96]. Finally, in subjects that do not respond to medical therapy, cardiac transplantation is an option, although it is rarely used in this condition.

## 7. Prognosis

In 2020, Aung et al. revealed in a meta-analysis that, when compared with dilated CM, LVNC patients have almost comparable risks of CV or death from all causes, ventricular arrhythmia, and thromboembolic complications [21,97]. Prognosis is mainly determined by the severity of LV systolic dysfunction; a low LVEF is the most significant indicator of an unfavorable result [21,97]. A recent study found that, as a group, compared with the general population, individuals with LVNC had worse overall survival rates, but those having a maintained LVEF and localized apical non-compaction responded better. Greater overall mortality was substantially correlated with the mid-non-compaction extent or basal non-compaction extent [98].

A recently published, large, multicenter French prospective registry found that, when comparing the results of 98 subjects having LVNC vs. 65 patients having dilated cardiomyopathy, an obvious trend toward poorer prognosis in LVNC vs. dilated CM was present for the studied patients with LV dysfunction. Although CV mortality was similar between LVNC and dilated CM, HF and/or rhythmic events (to a lesser degree) were more frequent in patients with LVNC, while embolic events occurred at the same rate [99].

Another recent retrospective multicentric study that included 200 patients with LVNC was aimed at evaluating the prognosis of different forms of LVNC. The subtype of dilated LVNC had the worst outcome, and independent factors for prognosis were age, LVEF < 50%, and ventricular tachycardia/fibrillation [100].

Long-term survival trials are warranted to assess the impact of different therapeutic strategies in individuals with LVNC [99]. Nevertheless, reverse remodeling, as in our patient, is associated with a better prognosis and lower mortality [101]. There is a wide spectrum of manifestations in LVNC, ranging from a strictly morphological perspective without hemodynamic impairment to a severe muscular disorder that is linked to a bad prognosis. The severity and mortality of LVNC in youth may be increased, as suggested by the current knowledge, particularly for those who present ventricular dysfunction within the first year of life [34].

## 8. Case Presentation

To clarify all aspects in the best way, our research exemplifies the situation of a 41-year-old male who had HF symptoms. LVNC CM was suspected at the time of transthoracic echocardiography and was subsequently confirmed during CMR processing. ARNI therapy was initiated, and the patient was prospectively observed for more than 12 months. Favorable remodeling and clinical outcomes were registered after including ARNI into the HF treatment. LVNC remains a heterogeneous CM, and although a favorable outcome is not commonly encountered, some patients respond well to therapy. The clinical presentation, paraclinical diagnosis, treatment strategy, and evolution are described. The patient filled out an informed consent form, and the study was carried out in conformity with the Declaration of Helsinki.

A 41-year-old male was hospitalized in February 2021 to the Cardiology Clinical Department of the Clinical County Emergency Hospital Oradea, Romania, with dyspnea at minimal exertion, dry cough, and fatigue. Symptoms occurred in the last 6 months and gradually worsened. On admission, he was already on a treatment that was initiated 3 months before, which used angiotensin converting enzyme (ACE) inhibitors (ramipril 5 mg/day), loop diuretics (furosemide 40 mg/day), mineral receptor antagonist (MRA) (spironolactone 50 mg/day), and beta-blockers (carvedilol 25 mg/day); however, his symptoms persisted. No family history of CM was present. On physical examination, we found a pulmonary stasis at the bases of the lungs, orthopnea, SO_2_ = 93% on ambient air, a blood pressure of 130/80 mmHg, an AV = 100 beats/min, an S3 and S4 gallop, and a systolic murmur grade of III/VI in the LV area.

**Figure 1:**
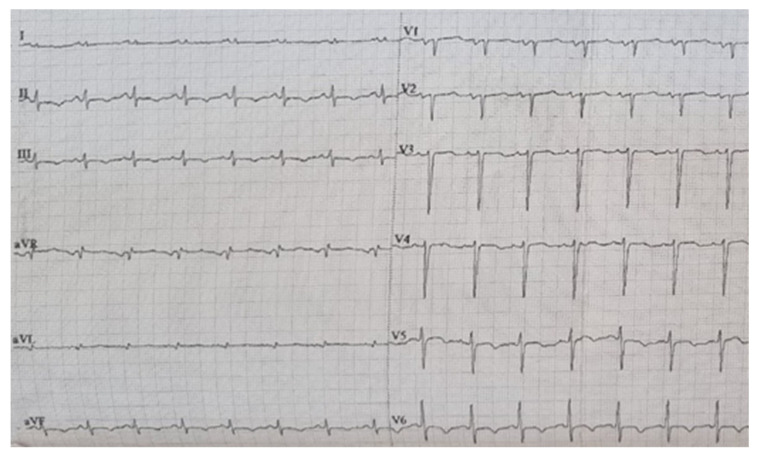
Twelve lead ECG.

Initial evaluation of the patient included twelve-lead electrocardiogram (ECG) and transthoracic echocardiography techniques. The ECG (performed with EDAN SE 1201, Edan Instruments Inc., Shangai International Holding Corp. GmBH (Europe) Hamburg, Gemany) showed the sinus rhythm, a heart rate of 90 beats/min, diffuse ST-T changes, T negative waves in inferior and lateral leads, and a flattened T wave in the rest of the leads (Figure 1, own archive of the last author).

**Figure 2:**
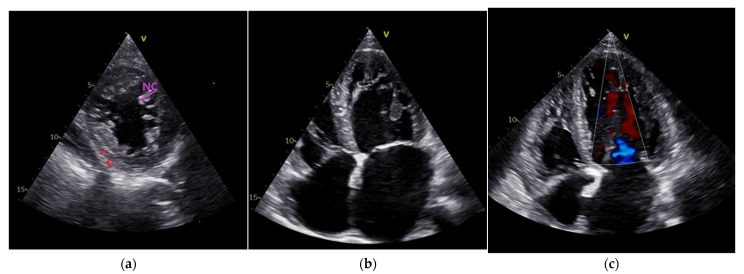
(**a**) Transthoracic echocardiography. Parasternal short-axis view. Non-compacted–NC/Compacted-C ratio > 2; (**b**) Apical 4 chamber view. Trabeculations at the apex and lateral wall of the LV, dilated left atrium. (**c**) Transthoracic echocardiography. Color flow in the intertrabecular recesses.

Transthoracic echocardiography (Figure 2, own archive of the last author), performed using VIVID E 95 (GE Vingmed Ultrasound AS, Horten, Norway), showed a moderately dilated LV with a morphologic image of a two-layered myocardium, which had trabeculations at the apex and in the mid-area of both the lateral anterior/inferior walls (Figure 2a,b). The ratio between the non-compacted/compacted myocardium at end systole in the short-axis perspective was 2.1. Color flow was present in the profound intertrabecular recesses (Figure 2c).

The contraction of the LV was severely altered, and diffuse hypokinesia was present, which was accentuated at the trabeculated area level. The left atrium (LA) was severely dilated. A spontaneous contrast was present in the LA and LV. Moderate mitral regurgitation due to LV dilatation was present. Compared with the biplane Simpson method baseline, the LVEF was reduced, and the LV end systolic and end-diastolic volumes were increased (Table 1). A tissue Doppler revealed decreased velocities at the level of the septal and lateral annulus. Examination of the LV diastolic function revealed a restrictive filling pattern of mitral diastolic inflow with an E/e′ ratio = 15. The LVEF and global longitudinal strain were lower in the speckle tracking echocardiography results (35% and −9.2%). The characteristic of a decreased LV twist motion of 2.6 (determined using the difference between the peak rotation at the level of base and the apex in the short-axis view) for LVNC was found. The right ventricle had increased apical trabeculations but normal fractional area variations and a tricuspid annular plane systolic excursion (difficult to differentiate from the normal variant in the highly trabeculated right ventricle). The tricuspid regurgitation was medium, the inferior vena cava was dilated with diminished inspiratory collapse, and the systolic pressure in the pulmonary artery was 60 mmHg. A transthoracic echocardiographic examination was strongly suggestive of a LVNC CM with a dilation of the LV and a depressed ejection fraction. The morpho-functional phenotype for our patient according to the MOGE(S) system for cardiomyopathies is M LVNC-D, LVNC with LV dilatation, and dysfunction. Holter ECG (BTL-08 Holter H600, BTL Industries Ltd., Cleveland, United Kingdom) monitoring for 24 h showed ventricular premature beats at a percentage of 3%. Laboratory tests revealed an elevated NT-proBNP level (Table 2).

**Figure 3:**
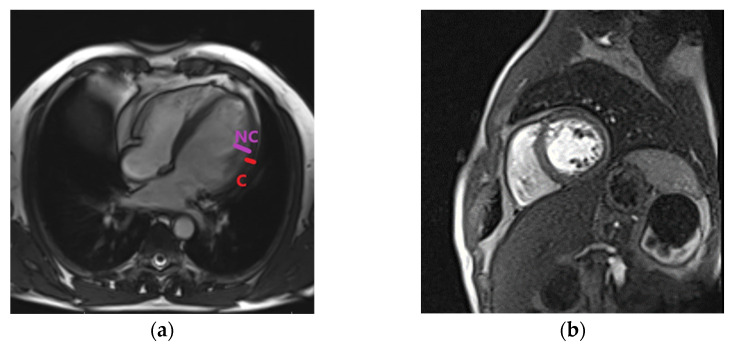
Cardiac magnetic resonance. (**a**) Left ventricular non-compaction in four chamber view. NC, non-compacted; C, compacted LV wall. (**b**) Left ventricular non-compaction in short-axis view.

A coronarography showed normal epicardial coronary arteries and excluded the ischemic etiology of HF. Genetic tests were not available for our patient. The patient was scheduled for a CMR evaluation and a further clarification of the diagnosis. HF treatment was administered in accordance with the ESC guidelines. ACE inhibitors were replaced after a washout period of 36 h with ARNI- sacubitril/valsartan at 100 mg/day, which was titrated to 200 mg/day after 2 weeks and an increased dosage of up to 400 mg/day after another month. Loop diuretics, MRA, and betablockers were continued, and anticoagulants were also associated. SGLT-2 inhibitors were not prescribed because at that time, they were not available in our hospital and were not sustained on free prescription by the healthcare system. There was a significant clinical improvement at 6 months after discharge; the patient was in the NYHA class I, and no symptoms were present. CMR processing was performed (using Siemens Magnetom_Essenza 1.5 T., Siemens Shenzen Magnetic Resonance LTD, Shenzen, China) at this time, and it confirmed the diagnosis of LVNC by identifying trabeculations that were located at the apex and medial levels of the anterior and lateral walls of the LV. The ratio between the non-compacted and compacted layers was 2.3 during diastole at the level of the lateral wall, fulfilling the Petersen criteria for diagnosis (Figure 3, own archive of the last author).

**Figure 4:**
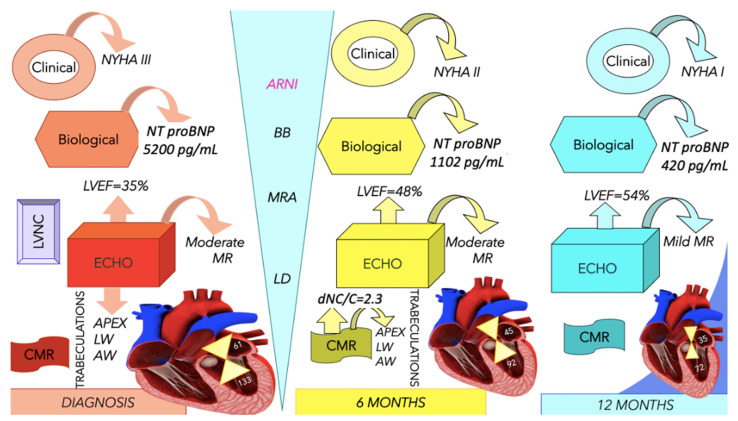
Elements of diagnosis for left ventricle non-compaction and the evolution of the presented case after therapy initiation. The numbers from the heart diagram illustrate the values of the indexed left atrial volume (mL/m^2^) and end-systolic volume of the left ventricle (mL); there is an improvement of the mentioned parameters after treatment initiation, and the clinical and biological elements expressed the same evolution. ARNI, angiotensin receptor neprilysin receptor; AW, anterior wall; BB, beta-blockers; CMR, cardiac magnetic resonance imaging; ECHO, echocardiography; LD, loop diuretics; LVEF, left ventricle ejection fraction; LVNC, left ventricle non-compaction; LW, lateral wall; MR, mitral regurgitation; MRA, mineralocorticoid receptor antagonists; dNC/C, diastolicnon-compacted/compacted ratio; NT-proBNP, N-terminal pro-b type natriuretic peptide; NYHA, New York Heart Association.

The LV was dilated, but significant reverse remodeling was observed at the time of echocardiography and CMR. LV and LA volumes were diminished compared with the initial evaluation, but contractility and LVEF were significantly improved. LGE was not detected in the CMR. After one year, the patient was free of symptoms, and a further improvement was observed at the time of echocardiography regarding the ejection fraction and left heart volumes (Table 1); however, increased trabeculations persisted. Figure 4 describes an algorithm used for the diagnosis of LVNC and illustrates the evolution of several parameters after treatment initiation. The clinical, biological, and echocardiographic elements showed a significant improvement, especially after 12 months of treatment.

Although it is not common, a favorable response, namely reverse cardiac remodeling, and clinical improvement, was observed after optimization of the treatment by adding ARNI to HF therapy.

The prognosis for LVNC is unpredictable due to the disorder’s significant heterogeneity. The important reverse remodeling observed in this patient was one of the main reasons that determined us to report the case. A similar improvement was observed after introduction of ARNI therapy in a few other LVNC case reports [63,65].

It is known that ARNI therapy that is initiated as early as possible can lead to greater cardiac reverse remodeling benefit in HF patients, having a reduced ejection fraction vs. angiotensin receptor blockers or angiotensin-converting enzyme suppressors. A recent systematic study that included a large number of patients with HF with reduced EF revealed that ARNI treatment was linked to an amelioration of the EF (+5.11%, 95% CI 4.06 to 6.16) and LV dimensions compared with patients who followed a treatment with angiotensin receptor blockers or angiotensin-converting enzyme suppressors [68].

The impressive reverse remodeling appeared in the context of an up-titration of the drug to the dose of 400 mg as recommended by the guidelines [102].

However, the magnitude of reverse remodeling is variable across patients. Several characteristics that were present in our patient were identified as predictors of reverse remodeling in various studies in patients with HF and reduced EF and in patients with LVNC. The etiology and duration of HF are factors that can influence outcome. The increase in LVEF is more important in patients with non-ischemic or new-onset HF (≤12 months) [103,104]. In an early and reversible stage of the disease, sacubitril/valsartan can prevent global cardiac remodeling, and this was sustained by the results of the PIONEER-HF trial (Comparison of Sacubitril/Valsartan Versus Enalapril on Effect on NT-proBNP in Patients Stabilized From an Acute Heart Failure Episode) [105].

The absence of a left bundle branch block is another independent predictor of reverse remodeling in cohort studies in patients with HF with reduced EF [106]. LV contractions are dyssynchronous, and inefficient, functional mitral regurgitation and reduced stroke volume are encountered in patients with left bundle branch blocks. The absence of myocardial fibrosis, as assessed using LGE CMR, in our patient is also correlated with reverse remodeling and a good prognosis [55,107]. Furthermore, an important decrease in NT-proBNP level is associated with greater improvements in the LVEF and a more important reduction of LV volumes [102]. Additionally, a younger age and a sinus rhythm identify a subgroup of patients with a more likely reverse LV remodeling [104]. All of these features that are associated with reverse remodeling were present in our patient.

## 9. Conclusions

The availability of high-resolution imaging techniques and the current awareness of the disease contribute to the increased number of patients that have been diagnosed lately with this CM. The diagnosis of LVNC is based on multimodality imaging investigations that combine echocardiography and CMR, but the diagnostic criteria are still not uniformly defined. There is a need for a consensus on the diagnostic criteria to avoid under- and overdiagnosis. To prevent overdiagnosis, it is still difficult to distinguish the LVNC phenotype from that of the healthy heart. Additionally, the phenotypes of other cardiomyopathies have a similar genetic profile overlap, which therefore represents another significant issue. Evidence supporting the treatment strategies in LVNC is limited, and no specific guidelines are available. Prospective trials to assess the management, therapeutic approach, and outcomes in this disease are warranted, but due to the low prevalence of this form of CM, this will present a real challenge.

Defining the CM etiology is important in all new-onset HF patients for the close monitoring and prevention of further complications or in the case that some specific treatment is attainable. Although irreversible LV dysfunction is more common in LVNC cardiomyopathies, the presented case highlights that the optimization of HF therapy is associated with significant reverse cardiac remodeling and important clinical improvement in some patients.

## Figures and Tables

**Table 1:** Left ventricular reverse remodeling studies and case reports in LVNC.

Type of Study/ No. of Patients	LVNC Phenotype/ Associated Diseases	Treatment	Reverse Remodeling Main Findings	Ref.
Case study	Dilated Hypothyroidism	ARNI	LVEF increased by 29%, reduction of LV end-diastolic diameter by 7 mm	[63]
Case study	Dilated	ARNI beta-blockers diuretics, aldosterone antagonists	LVEF increased from 24% to 51% in 16 months, LV cavity decreased, diastolic function improved E/e’ decreased from >15 to 10–14; the ratio of non-compacted/compacted myocardium decreased	[65]
Retrospective/ 51	Dilated	3 betablockers 15 ACEi/ARB 33 dual therapies	88% had an improvement in LVEF by 16 ± 12% LV shortening fraction improved by 8 ± 9%	[12]
Prospective/ 23		ACEi and/or ARB and beta-blockers in addition to diuretics	39% had an absolute increase in LVEF > 10% at 6 months Regression of LVHT area showed significant correlations with the changes in LVEF	[39]
Prospective/ 20		CRT	60% responders vs. 28% with DCM	[43]
Prospective/11			All patients were responders Phase standard deviation was reduced from 89.5″± 14.2″ to 63.7″ ± 20.5″	[84]
Prospective/15			LVEF increased from 27.6 ± 5.5 to 39.1 ± 7.0% (*p* < 0.01) LV volumes did not change significantly	[85]
Systematic review/70			50% responders LVEF increased from 8 to 36% NYHA class improved	[86]
Case study		Carvedilol, lisinopril, furosemide	LVEF increased from 15–20% to 55% and LVEDV decreased from 210 mL to 145 mL at 1 year	[44]
Case study	Dilated Polyneuropathy	Biventricular pacemaker system	LV function improved, LV size decreased, LVHT could not be longer detected	[10]
Case study	Dilated/ Severe aortic regurgitation	Aortic valve replacement	Regression of LV dimensions and improvement of LVEF	[9]
Case study	Dilated	Standard HF treatment ICD-CRT	LVEF increased from 18 to 51%, morphologic features of LVNC become less clear	[45]
Case study		ACEi, beta-blockers, diuretics, aldosterone antagonists	LVEF increased from 19 to 47%, LV end-diastolic diameter decreased from 70 mm to 61 mm, resolution of non-compacted appearance	[46]
Case study		ICD-CRT	LVEF increased from <20% to 60% with almost complete resolution of LVHT	[47]

LVEF, left ventricular ejection fraction; ARNI, angiotensin receptor neprilysininhibitor; ACEi, angiotensin converting enzyme inhibitor; ARB, angiotensin receptor blocker; CRT, cardiac resynchronization therapy; LVHT, left ventricle hyper trabeculation; LVEDV, left ventricular end-diastolic volume; DCM, dilated cardiomyopathy; ICD-CRT, internal cardioverter defibrillator-cardiac resynchronization therapy; HF, heart failure.

**Table 2:** Evolution of echocardiographic parameters and NT-proBNP level.

Parameters	Baseline	6 Months	12 Months
LVEDV (mL)	202	177	152
LVESV (mL)	133	92	72
LVEF (%)	35	48	54
LAVI (mL/m^2^)	61	45	35
Mitral regurgitation	Moderate	Moderate	Mild
NT-proBNP (pg/mL)	5200	1102	420

LVEDV, left ventricular end-diastolic volume; LVESV, left ventricular end-systolic volume; LVEF, left ventricular ejection fraction; LAVI, left atrial volume index; NT-proBNP, N-terminal Pro B-type natriuretic peptide.

## Data Availability

Data are contained within the article.
